# Quality of life and associated predictors in Palestinian medical students: a cross-sectional study

**DOI:** 10.1080/10872981.2025.2576125

**Published:** 2025-10-29

**Authors:** Amer Rahhal, Mohammad Isbeih, Hamid Ghanem, Nour Jaber, Shahd Idais, Ibrahim Amer Ghannam, Maha Nubani Husseini, Maha Nahal

**Affiliations:** a Physiology and Pharmacology Department, Faculty of Medicine, Al-Quds University, Jerusalem, Palestine; b Faculty of Medicine, Al-Quds University, Jerusalem, Palestine; c Medical Research Club, Faculty of Medicine, Al-Quds University, Jerusalem, Palestine; d Department of Medical Laboratory Sciences, Faculty of Health Professions, Al-Quds University, Jerusalem, Palestine; e Department of Public Health & Nutrition, Faculty of Public Health, Al-Quds University, Jerusalem, Palestine; f Department of Midwifery & Master Program of Maternal Child Health, Faculty of Health Professions, Al-Quds University, Jerusalem, Palestine

**Keywords:** Quality of life, medical students, social relationships, psychological well-being, physical health, preclinical phase, clinical phase

## Abstract

Medical students experience tremendous stressors related to the demanding nature of medical education programs, which can negatively impact their health and overall quality of life (QoL). This particular study aimed to evaluate the QoL of medical students at Al-Quds University (AQU) in Palestine across various academic years and demographic variables, concerning physical health, psychological well-being, social relationships, and environmental conditions. A cross-sectional comparative study involving 522 medical students representing all six academic years was conducted from May to July 2024. QoL was assessed via the culturally adapted Arabic version of the World Health Organization Quality of Life (WHOQOL-BREF) questionnaire. Demographic variables (gender, age, place of residence, number of bedrooms) and academic (academic year) variables were examined as correlates of QoL domains. Descriptive statistics, independent *t*-tests, and one-way ANOVA were applied, and linear regression was used to identify predictors. A *p*-value of ≤0.05 was considered statistically significant. Social relationships were the highest-rated domain (M = 3.58, SD = 0.862), followed by physical health (M = 3.38, SD = 0.721), environment (M = 3.28, SD = 0.638), and psychological conditions (M = 3.12, SD = 0.693). Gender differences emerged, with males reporting better physical health (*p* = 0.002). Linear regression further identified academic level as a key predictor of physical health (*p* < 0.001). Medical students at AQU have shown resilience in their social relationships. However, concerns arise from their lower psychological health and limited access to recreational opportunities. These findings highlight the importance of targeted institutional strategies that address mental health and foster supportive environments. Ultimately, this approach aims to enhance student well-being and create a more balanced and sustainable quality of life for students.

## Background

Quality of life (QoL) is an individual's perception of his or her position in life, and is influenced by his or her environment, culture, expectations, standards, and concerns [1]. The QoL of medical students has become a growing global concern, particularly in developing countries, where significant hardships aggravate the stress of medical education [2–4]. Stress among medical students begins upon entering medical colleges, driven by limited resources, an intensive curriculum, demanding clinical training, and financial challenges [5–8]. On top of that, medical students face various physical, socioemotional, and family-related pressures that can significantly impact their physical and mental health, challenging their intentions to achieve a successful medical career [6,7]. These stressors, combined with substandard living conditions in Palestine and an ongoing occupation, can severely affect the QoL and academic performance of medical students in the region [5,8,9]. This heightens their vulnerability to low self-concept and compromised physical and mental health [10–12]. Therefore, medical educators must prioritize students' quality of life alongside their education to ensure overall well-being and academic success.

Previous studies have reported high rates of anxiety and depression among medical students [13,14]. In Middle Eastern countries, the prevalence of depression is particularly high, with rates varying across academic years [15]. Common associated factors are female gender, economic burdens, and academic clinical training pressures [15,16]. The sociodemographic characteristics that have also been associated with medical students' QoL are gender, marital status, employment, living, and housing conditions [17].

Understanding QoL is vital for university policymakers and educators to create strategies that support students' mental and physical health, and ensure academic and professional success [18]. Despite its critical importance, studies focusing exclusively on QoL domains among medical students in Palestine are limited. A study among Palestinian medical students showed high depression and anxiety rates, with academic stage, grade point average (GPA), mental health, and religious commitment linked to outcomes. These findings highlight the need for targeted support [2]. Furthermore, Gaza medical students face high levels of mental distress and poor sleep, with low income and inadequate sleep as key predictors, highlighting the urgent need for targeted support [19]. Additionally, among Palestinian university students, QoL was shaped by lifestyle and demographics; better sleep, physical activity, and higher income improved well-being, while poor sleep and heavy study loads reduced it, underscoring the need for supportive programs [20]. However, this is expected to be the first study to assess the QoL of medical students across all six years of study at AQU, Palestine. Accordingly, this study aimed to evaluate how students perceive and describe their QoL, as it is crucial in enhancing their university experience and shaping their future career choices. Specifically, it focused on the impact of academic years and demographic factors on QoL, examining physical health, psychological well-being, social relationships, and environmental conditions.

## Methods

### Study design

A comparative cross-sectional study was conducted in May 2024 among male and female students enrolled in the MD program at the Faculty of Medicine at AQU, in Jerusalem, Palestine. The primary objective was to assess the quality of life (QoL) among medical students across different academic levels. The AQU Research Ethics Committee (Ref No: 378/REC/2024) granted ethical approval for the study.

### Setting

The six-year Doctor of Medicine (MD) program at AQU's Faculty of Medicine in Palestine provides a comprehensive education preparing students for modern medical practice. The first three years comprise the preclinical phase, which focuses on foundational medical sciences. In the clinical phase (years four to six), students engage in hands-on training at affiliated hospitals and health centers, applying theoretical knowledge in real-world settings and developing essential clinical and diagnostic skills. With approximately 350 students per academic year, the program also fosters a collaborative and supportive learning environment.

### Participants and eligibility criteria

All medical students enrolled at AQU for the 2023/2024 academic year were eligible to participate, regardless of age, gender, or other demographic factors. The broad inclusion criteria aim to foster a comprehensive understanding of quality of life across different academic years and demographic backgrounds. We ensured adequate representation, enabling comparisons of variations among subgroups, such as year of study, gender, and place of residence. At the time of data collection, the total student population was approximately 2200. The only exclusion criterion was the refusal of students to provide informed consent. Participation was voluntary, and no incentives were offered.

### Sample size and sampling strategy

The target sample size was 522 students, calculated using G*Power version 3.1.9.7 for a one-way ANOVA (fixed effects, omnibus) with six groups representing the academic years. We set the effect size at f = 0.20, the significance level (*α*) at 0.05, and the desired power at 0.90, which yielded a minimum required sample size of 420 students. To account for potential non-response and incomplete questionnaires, the sample size was increased by approximately 25%, resulting in the final target of 522 students. To ensure adequate representation across all academic years, a proportional quota sampling approach was used. The quotas for each year were based on their proportion in the total student population: first year (*n* = 101), second year (*n* = 100), third year (*n* = 116), fourth year (*n* = 61), fifth year (*n* = 86), and sixth year (*n* = 58).

### Variables and measurement tools

The primary outcome variable was quality of life (QoL), assessed across four domains: physical health, psychological health, social relationships, and environment. Additional sociodemographic data were collected from students, including age, sex, academic year or level, parents' education, and place of residence. QoL was measured using the WHOQOL-BREF, a 26-item instrument that has been validated in multiple languages and populations [19–21]. It demonstrates acceptable validity, internal consistency, and test-retest reliability. The instrument also includes two items that assess overall perceptions of QoL and general health. Responses were recorded on a 5-point Likert scale for the four domains, where 1 indicates very poor/very dissatisfied/never and 5 indicates very good/very satisfied/always, with higher scores reflecting better QoL.

To enhance cultural appropriateness, two culturally sensitive items were removed following expert consultation. In this study, we utilized the Arabic version of the WHOQOL-BREF, which has been previously validated in Arab populations [21]. The reliability analysis of the QoL questionnaire in this study demonstrated strong internal consistency across all domains, with Cronbach's *α* values of 0.802 for physical health, 0.813 for psychological health, 0.746 for social relationships, and 0.815 for the environment. The overall Cronbach's *α* value was 0.897, indicating that the items within each domain consistently measure the same underlying concept.

### Pilot testing

A pilot test was conducted among a small group of students to ensure clarity, cultural appropriateness, and reliability of the questionnaire. Feedback from the pilot informed minor revisions that were corrected before full distribution.

### Data collection procedures

Data collection occurred one month before scheduled examinations to minimize the impact of exam-related stress on responses. The survey was administered via Google Forms and distributed electronically. Students were instructed to complete the questionnaire privately and at their convenience. Written informed consent was obtained electronically before participation. Completed responses were automatically received via email. Recruitment was facilitated using a convenience sampling technique. An invitation was posted on the official AQU medical students' social media platforms, containing a link to the online survey.

### Bias minimization

To reduce selection bias, participation was open to all students across academic years, with proportional quota sampling to ensure balanced representation. The timing of data collection was carefully chosen to limit stress-related response bias. The use of a validated Arabic tool minimized measurement bias.

### Statistical analysis

Data were analyzed using IBM SPSS Statistics v27 and Jamovi v2.3 (R-based). Index variables for each QoL domain were created by calculating the mean score of the items within that domain. Internal consistency was assessed using Cronbach’s alpha. A significance level of *p* < 0.05 was used to determine statistical significance.

The following statistical analyses were performed:

• Chi-square tests to assess associations between categorical variables (e.g., gender and QoL domains).
• One-way ANOVA to compare QoL scores across academic years.
• Independent-sample *t*-tests to compare QoL between pre-clinical and clinical phases.
• Linear regression analysis to identify predictors of QoL, such as age, academic level, and number of bedrooms.
• Pearson correlation coefficients to investigate the relationships between the continuous variables.

## Results

### A. Participant characteristics

The medical students were predominantly female, with 345 (66.1%) females and 177 (33.9%) males. Most of the participating students were single (98.8%), and had a median age analysis of quality of life (QoL) factors across various demographic variables of 21 years (IQR: 20−22, range: 17−32). The students were fairly evenly distributed across academic years, with a total of 116 (22.2%) students in the third year and only 58 (11.1%) in the sixth year. The majority of the medical students were from Jerusalem (37.7%), followed by Hebron (16.5%) and Ramallah (16.1%). They mostly resided in cities (54.0%) and villages (43.1%). The sociodemographic characteristics of the participants are shown in Table 1.

**Table 1.** Sociodemographic characteristics of the participating medical students (*N* = 522).

Sociodemographic		*N* (%)
**Gender**	Female	345 (66.1%)
	Male	177 (33.9%)
**Social status**	Single	515 (98.8%)
	Married	6 (1.2%)
**Governorate**	Bethlehem	61 (11.7%)
	Hebron	86 (16.5%)
	Jerusalem	197 (37.7%)
	Ramallah	84 (16.1%)
	Israel	57 (10.9%)
	Others	37 (7.1%)
**Place of residence**	Camp	15 (2.9%)
	City	282 (54.0%)
	Village	225 (43.1%)
**Mother education**	Primary	21 (4.0%)
	Secondary	122 (23.4%)
	University	315 (60.5%)
	Post Graduate	63 (12.1%)
**Father education**	Primary	36 (7.0%)
	Secondary	159 (30.8%)
	University	219 (42.4%)
	Post Graduate	102 (19.8%)
**Academic level**	1^st^ Year	101 (19.3%)
	2^nd^ Year	100 (19.2%)
	3^rd^ Year	116 (22.2%)
	4^th^ Year	61 (11.7%)
	5^th^ Year	86 (16.5%)
	6^th^ Year	58 (11.1%)

### B. Quality of life

Analysis of the four QoL domains shown in Table 2 revealed that social relationships were rated highest on average (M = 3.58, SD = 0.862), followed by physical health (M = 3.38, SD = 0.721), the environment (M = 3.28, SD = 0.638), and psychological conditions (M = 3.12, SD = 0.693). Social relationships presented the greatest variability in response, whereas the environment presented the least variability. All the domains nearly or entirely spanned the full possible range [1–5], indicating diverse experiences across the sample. The study revealed a range of perceptions across four domains of quality of life. In the physical health domain, 176 participants (33.7%) reported that extreme pain significantly affected their daily activities, whereas 292 participants (55.9%) required medical interventions for these activities at an extreme level. Nevertheless, 289 (55.4%) medical students reported the ability to move around as good or very good. In the psychological health domain, 267 (51.1%) of the participants reported only moderate enjoyment of life, whereas a separate 235 (45.1%) felt that their lives were extremely meaningful, indicating varied psychological experiences. Additionally, 325 (62.2%) expressed satisfaction with themselves. In the social relationships domain, 320 (61.3%) respondents reported satisfaction with their social interactions, including personal relationships and friend support. The environmental domain showed both positive and concerns. Most participants 444 (85.1%) felt safe, and 389 (74.6%) considered their environment healthy. However, 275 (52.7%) of the participating medical students reported having limited recreational opportunities.

**Table 2.** Quality of life (WHOQOL-BREF) of medical students at Al-Quds University (*N* = 522).

Quality of life	Mean (SD)	Median	Minimum	Maximum
Physical health	3.38 (0.721)	3.43	1.29	5
Psychological conditions	3.12 (0.693)	3.17	1	4.83
Social relationships	3.58 (0.862)	4	1	5
Environment	3.28 (0.638)	3.25	1	5

### C. Age, housing, and residence effects on quality of life

Multivariate regression analysis (Table 3) examining predictors of the four WHOQOL-BREF domains revealed distinct patterns across domains. Age and academic level emerged as consistent positive predictors, particularly for physical health (*β* = 0.187, *p* < 0.001; *β* = 0.181, *p* < 0.001, respectively) and social relationships. Male gender was significantly associated with higher physical health scores (*β* = 0.133, *p* = 0.002), but not with other domains. The number of bedrooms in the household, reflecting housing quality or socioeconomic status, had a significant positive effect across all domains except psychological, and was the strongest predictor of environmental quality (*β* = 0.274, *p* < 0.001). Residence place was negatively associated with the psychological (*β* = –0.120, *p* = 0.006), social (*β* = –0.113, *p* = 0.010), and environmental scores (*β* = –0.213, *p* < 0.001), suggesting a possible urban‒rural disparity. Parental education, particularly fathers' and mothers' education, significantly predicted better environmental quality, whereas family composition variables (e.g., siblings, household crowding) had negligible effects across outcomes.

**Table 3.** Multivariate linear regression coefficients for predictors of quality of life domains among medical students. Standardized beta coefficients (*β*) and significance levels (*p*-values) for predictors across four WHOQOL-BREF domains.

Predictor	Physical Health *β* (*p*)	Psychological *β* (*p*)	Social Relations *β* (*p*)	Environment *β* (*p*)
Age	**0.187 (<0.001)**	0.080 (0.069)	0.094 (0.032)	0.062 (0.156)
Academic level	**0.181 (<0.001)**	0.082 (0.060)	0.097 (0.026)	0.062 (0.156)
Sex (male)	**0.133 (0.002)**	0.025 (0.570)	–0.021 (0.636)	–0.009 (0.836)
No. of bedrooms	0.105 (0.017)	0.082 (0.060)	0.102 (0.020)	**0.274 (<0.001)**
Residence place	–0.067 (0.127)	**–0.120 (0.006)**	**–0.113 (0.010)**	**–0.213 (<0.001)**
No. of siblings	–0.076 (0.083)	–0.072 (0.100)	–0.048 (0.271)	**–0.134 (0.002)**
Family members sharing home	–0.056 (0.201)	–0.042 (0.341)	–0.011 (0.795)	–0.084 (0.054)
Father's education	0.086 (0.052)	0.047 (0.285)	0.032 (0.469)	**0.215 (<0.001)**
Mother's education	0.051 (0.244)	0.040 (0.363)	0.060 (0.171)	**0.157 (<0.001)**
Mother's job	–0.073 (0.099)	–0.023 (0.598)	–0.023 (0.602)	–0.103 (0.019)
Governorate	0.030 (0.500)	0.068 (0.121)	0.040 (0.360)	0.110 (0.012)
Father's job	–0.041 (0.352)	0.036 (0.415)	–0.025 (0.570)	–0.001 (0.980)

Bold values indicate statistical significance (p < 0.05).

### D. Correlations

All four QoL main factor groups were significantly positively correlated with each other (all *p* < 0.001). The strongest correlation was between physical health and psychological conditions (*r* = 0.607), whereas the weakest correlation was between physical health and social relationships (*r* = 0.352). Age was positively correlated with physical health (*r* = 0.187, *p* < 0.001) and social relationships (*r* = 0.094, *p* = 0.032). The number of siblings was negatively correlated with the environment (*r* = −0.134, *p* = 0.002). The number of bedrooms in the home was positively correlated with the environment (*r* = 0.274, *p* < 0.001), physical health (*r* = 0.105, *p* = 0.017), and social relationships (*r* = 0.102, *p* = 0.020). No significant correlations were found between QoL factors and the number of family members sharing the same home.

### E. Difference in medical students' QoL based on sociodemographic characteristics

**Figure 1.**
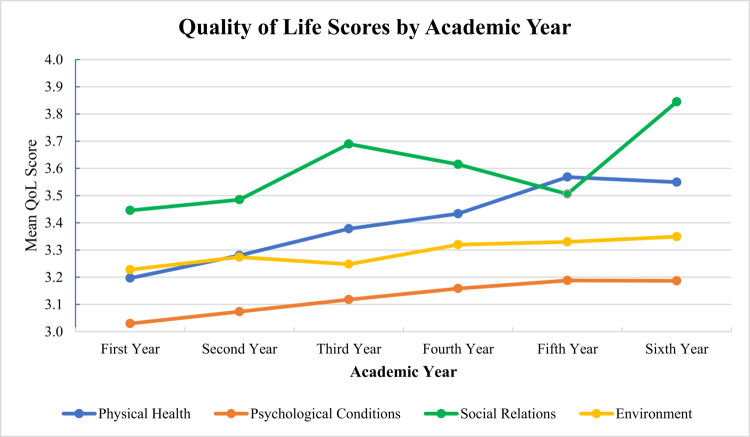
Progressive improvement in quality of life across academic levels: a general upward Pattern in physical health, psychological well-being, and environmental factors suggests enhanced student well-being over time, with social relationships showing variability before peaking in the final year.

1. **Gender:** The study revealed a significant difference between male and female students in reporting the quality of their physical health (*p* = 0.002). This is supported by the mean and standard deviation values (M = 3.514, SD = 0.737) for males and (M = 3.313, SD = 0.703) for females. No statistically significant differences were observed in the other quality health domains related to gender differences.
2. **Academic years:** The results indicate a general upward trend in QoL scores across academic years, as shown in Figure 1. The scores for physical health (*p* = 0.003), psychological conditions, and the environmental domain progressively increased from the first year to the sixth year, suggesting an overall increase in students' well-being as they advanced academically. Notably, social relationships (*p* = 0.037) scores exhibit some variability, peaking in the third year, declining slightly in the fourth and fifth years, and then reaching their highest level in the sixth year. This pattern may reflect evolving social integration and support networks among students. These findings suggest that improved academic adaptation, resilience, and coping strategies have developed over time.
3. **Governorate:** Environment scores were significantly different from those of the on governorate (*p* = 0.036), with Bethlehem reporting the lowest mean score (M = 3.131, SD = 0.656) and those of students who reside in Israel reporting the highest score (M = 3.463, SD = 0.690).
4. **Parental education:** Both the mother's education (*p* < 0.001) and the father's education (*p* < 0.001) were strongly associated with the environmental domain of QoL. Participants with more educated parents reported better environment scores. The psychological conditions significantly differed according to the mother's education level (*p* = 0.032).
5. **Parental occupation:** Father's job (*p* < 0.001) and mother's job (*p* = 0.038) were also significant factors. Those whose fathers held higher-status jobs reported better environmental QoL. No significant differences were found in QoL factors based on parents' jobs for domains other than the environment.
6. **Place of residence:** Students residing in camps reported the highest mean score for psychological conditions (M = 3.278, SD = 0.619, *p* = 0.023) as well as for social relationships (M = 3.867, SD = 0.719, *p* = 0.036). However, students who lived in cities reported the highest scores for environmental quality (M = 3.409, SD = 0.632, *p* < 0.001). On the other hand, villages scored the lowest across all the QoL domains.

### F. Means of transportation

Overall satisfaction was split: 40.2% of the students reported being either satisfied or strongly satisfied with their means of transportation, whereas 22.6% remained neutral. However, dissatisfaction was notable, with 37.2% of the students expressing dissatisfaction (22.4% dissatisfied and 14.8% strongly dissatisfied). Dissatisfaction was most pronounced in the third and sixth years, where dissatisfaction rates peaked at 39.7% and 51.7%, respectively. The sixth-year cohort had the highest proportion of strong dissatisfaction at 22.4%. In contrast, satisfaction was highest among first and fourth-year students, with 33.7% and 32.8%, respectively, reporting being satisfied with their transportation options. This may be explained by the fact that transportation to the university is a new experience for first-year students, making them more optimistic about it. Additionally, for fourth-year students, who begin their clinical training, transportation experiences vary depending on the proximity of hospital locations to their residences.

### G. Preclinical vs clinical years

The statistical analysis revealed several significant differences between the two groups across multiple dimensions of well-being and functional capacity. A significantly greater proportion of the clinical phase students reported enjoying life ‘very much’ than did the preclinical phase students (41.5% vs. 30.9%, *p* = 0.014). Additionally, the clinical group exhibited a greater frequency of negative emotions, with 45.4% reporting feeling negative emotions ‘very much’ compared with 35.0% in the preclinical group (*p* = 0.018). Similarly, participants in the clinical group showed a reduced level of dissatisfaction with work capabilities, with fewer individuals reporting being ‘strongly dissatisfied’ (4.4% vs. 12.3%, *p* = 0.002). In terms of physical energy, a significantly greater proportion of the clinical group reported having no energy ‘at all’ to engage in daily activities (3.4% vs. 9.1%, *p* = 0.012). Furthermore, the clinical group demonstrated improved satisfaction with personal relationships, with 52.2% reporting satisfaction compared with 43.2% in the preclinical group (*p* = 0.045).

## Discussion

The QoL of medical students is critical to their academic performance, mental well-being, and future professional success [22]. This study provides an in-depth examination of these aspects across different academic years, focusing on physical health, psychological well-being, social relationships, and environmental conditions. The findings not only confirm prior research [21,22] but also offer new insights specific to the Palestinian context, emphasizing the necessity for targeted interventions in medical education.

The Arabic version of the WHOQOL-BREF instrument has proven reliable and valid for assessing QoL among Palestinian medical students. This aligns with prior validation studies [1,23], reinforcing its applicability in diverse cultural contexts. The robust psychometric properties of this instrument provide a solid foundation for future QoL assessments within Arabic-speaking student populations.

The study's demographic profile, showing a predominance of female students, is consistent with global trends in medical education [24]. This gender distribution has significant implications, particularly in the context of gender-based disparities in medical training and stress management. The median age of 21 years aligns with the expected norms for medical education, confirming the representativeness of the sample. Additionally, the geographic diversity of students highlights socioenvironmental influences on QoL, mirroring similar studies in the Middle East [21].

Interestingly, the highest-rated QoL domain was social relationships, indicating that medical students benefit from strong peer and familial support systems within Palestinian culture. These support networks play crucial roles in fostering resilience and helping students navigate the challenges of an overwhelming context [25]. These results are also consistent with the literature, which suggests that supportive social networks mitigate academic stress [14]. However, while students report high levels of satisfaction with relationships, the academic rigor of medical education can still impose significant emotional strain, necessitating continuous assessment and enhancement of peer-support programs. On the other hand, the physical health of medical students has received moderate ratings, with notable concerns regarding the effect of extreme pain on daily activities. This suggests a correlation between academic workload and physical strain [26]. Furthermore, the regression findings complement other analyses by highlighting the multidimensional nature of QoL among medical students. Reinforce the influence of both structural and individual-level factors. Notably, age and academic progression are consistently associated with better physical and social well-being, supporting prior findings that resilience and adaptive capacity improve with experience and academic maturity [14,27]. The number of bedrooms – a proxy for living space and socioeconomic status – was a strong positive predictor, particularly in the environmental domain. Aligning with previous evidence that material living conditions shape perceptions of safety, autonomy, and access to resources [28]. Conversely, psychological QoL was least explained by demographic or socioeconomic variables, which is consistent with the literature suggesting that internal stressors, academic demands, and coping styles play a more dominant role in mental health outcomes among medical students [29]. Residence location showed a consistently negative association across multiple domains, indicating possible disparities in access to services, safety, or community support [30]. The strong link between parental education and environmental quality further underscores the long-term impact of social capital and educational privilege on students’ lived experiences [31]. Collectively, these findings support a holistic approach to student well-being that incorporates not only academic interventions, but also improvements in housing, mental health support, and campus infrastructure [32,33]. In addition to structural and systemic factors, the observed gender disparities in physical health, where males reported higher scores, highlight another critical dimension of student well-being. This aligns with studies indicating that female medical students experience more physical discomfort and stress-related symptoms [34]. These findings highlight the need for ergonomic interventions, structured exercise programs, and stress reduction strategies within medical institutions. In addition, the psychological QoL of the medical students in this study was the lowest-rated domain. This might reflect the high prevalence of stress and emotional distress among medical students related to the condensed medical education program. Furthermore, it can be linked to the ongoing political conflict in the Palestinian context, which impacts every aspect of the student's life [5,25]. Compared with male students, female students reported significantly more negative emotions, corroborating research suggesting that women in academic settings are more prone to psychological distress [35]. Although our findings align with global findings on stress among medical students, it is important to consider specific stressors faced by Palestinian students, such as political instability, economic pressures, and cultural expectations. Research indicates that political instability, including violence and commuting challenges due to checkpoints, significantly impacts Palestinian medical students' ability to concentrate and perform academically [36]. Additionally, economic pressures and cultural expectations contribute to elevated levels of anxiety and depression among these students [37]. This finding underscores the necessity for gender-sensitive mental health interventions, including accessible counseling services and resilience training. The students from Bethlehem reported the lowest environmental QoL scores, indicating that geographic disparities impact perceptions of infrastructure and living conditions. This finding aligns with studies linking regional development levels to student well-being [38]. Additionally, higher parental education was associated with better environmental QoL and improved psychological conditions, reinforcing the socioeconomic determinants of student well-being [39]. Significant variation in physical health across academic years was observed, with senior students reporting better scores. This suggests that students in advanced years may develop better coping mechanisms and adapt to the physical demands of medical training [40]. Conversely, high sleep dissatisfaction among preclinical students aligns with studies linking early-stage academic stress with poor sleep quality [41]. These findings emphasize the need for academic policies promoting work‒life balance, particularly for early-year students.

Although students who had begun clinical training reported higher levels of life enjoyment and relationship satisfaction compared to those who had not yet started. They also experienced negative emotions. This dual outcome – higher life enjoyment paired with increased negative emotions – mirrors findings that clinical exposure boosts professional fulfillment while also heightening emotional exhaustion [42]. The reported negative emotions in this study may be linked to the challenges Palestinian students face when traveling to clinical settings across different governorates. Transport difficulties significantly impact the Palestinian population due to political instability and the Gaza conflict that escalated in October 2023 [5], affecting students’ commutes to the university campus and the clinical training sites. These findings demonstrate the need for structured emotional support and mentorship during clinical training. Furthermore, strong correlations between physical health and psychological conditions validate the well-established link between physical and mental health [43]. Additionally, environmental factors, such as living conditions and available infrastructure, significantly influence QoL, highlighting the role of socioeconomic stability [44]. These findings suggest that efforts to improve QoL should take a holistic approach, simultaneously addressing physical, psychological, and environmental factors.

### Implications and recommendations

The findings of this study suggest several key interventions for improving medical students' QoL:

1. The medical education curriculum should incorporate a comprehensive resilience program with structured mental health initiatives for all of the medical students, especially female students, who report greater psychological distress.
2. Addressing limited recreational opportunities can significantly improve environmental QoL.
3. Emphasis should be given to social support. This includes strengthening family awareness of student stressors, fostering peer networks and mentorship programs within medical schools, and ensuring institutional support through counseling and student wellness services. The Palestinian cultural context highly values community and family bonds, which can effectively mitigate psychological distress and foster resilience.
4. Strategies to improve sleep quality and manage workload stress should be promoted, particularly for preclinical students.
5. Ergonomic assessments, stress management training, and exercise programs should be integrated into medical curricula.
6. Providing additional resources and financial aid programs can help mitigate the impact of socioeconomic disparities on QoL.
7. Medical students require mentorship programs that offer both academic guidance and emotional support to help them navigate the challenges of clinical training.

## Limitations

The current study had several limitations. Despite its substantial sample size, the utilization of the validated WHOQOL-BREF instrument and the inclusion of students across all six academic years provide a comprehensive assessment of QoL. Nonetheless, it is a single-institution context; given that the study was confined to one medical school, findings may not comprehensively reflect the experiences of medical students across other Palestinian universities. Additionally, the self-reported data on QoL and associated factors were assessed using self-administered questionnaires, which may introduce reporting bias or social desirability bias. Finally, the context-specific stressors, political instability, and socioeconomic hardship in Palestine may limit the generalizability of results to students in different contexts.

## Conclusion

Medical students at AQU experience variable QoL across domains, with psychological well-being consistently lowest and social relationships highest. QoL was shaped by gender, parental education, residence, housing conditions, and stage of training. Senior students reported better QoL, while female students, those from rural areas, and those with poor housing reported lower scores, reflecting persistent disparities. Within the Palestinian context, political instability, economic strain, and transportation challenges intensify stress, particularly during clinical years. To address these concerns, medical schools should strengthen mental health support, mentorship, and resilience training, while fostering social and family networks. Prioritizing student well-being alongside academics is vital for preparing healthier, more resilient physicians.

## Data Availability

The datasets used in the current study are available from the corresponding author upon reasonable request.
